# Penta-EF-Hand Protein Peflin Is a Negative Regulator of ER-To-Golgi Transport

**DOI:** 10.1371/journal.pone.0157227

**Published:** 2016-06-08

**Authors:** Mariah Rayl, Mishana Truitt, Aaron Held, John Sargeant, Kevin Thorsen, Jesse C. Hay

**Affiliations:** Division of Biological Sciences and Center for Structural and Functional Neuroscience, The University of Montana, Missoula, MT, United States of America; The Chinese University of Hong Kong, HONG KONG

## Abstract

Luminal calcium regulates vesicle transport early in the secretory pathway. In ER-to-Golgi transport, depletion of luminal calcium leads to significantly reduced transport and a buildup of budding and newly budded COPII vesicles and vesicle proteins. Effects of luminal calcium on transport may be mediated by cytoplasmic calcium sensors near ER exits sites (ERES). The penta-EF-hand (PEF) protein apoptosis-linked gene 2 (ALG-2) stabilizes sec31A at ER exit sites (ERES) and promotes the assembly of inner and outer shell COPII components. However, in vitro and intact cell approaches have not determined whether ALG-2 is a negative or positive regulator, or a regulator at all, under basal physiological conditions. ALG-2 interacts with another PEF protein, peflin, to form cytosolic heterodimers that dissociate in response to calcium. However, a biological function for peflin has not been demonstrated and whether peflin and the ALG-2/peflin interaction modulates transport has not been investigated. Using an intact, single cell, morphological assay for ER-to-Golgi transport in normal rat kidney (NRK) cells, we found that depletion of peflin using siRNA resulted in significantly faster transport of the membrane cargo VSV-G. Double depletion of peflin and ALG-2 blocked the increased transport resulting from peflin depletion, demonstrating a role for ALG-2 in the increased transport. Furthermore, peflin depletion caused increased targeting of ALG-2 to ERES and increased ALG-2/sec31A interactions, suggesting that peflin may normally inhibit transport by preventing ALG-2/sec31A interactions. This work identifies for the first time a clear steady state role for a PEF protein in ER-to-Golgi transport—peflin is a negative regulator of transport.

## Introduction

The ER-to-Golgi interface is the busiest vesicle trafficking step, transporting up to one-third of all eukaryotic proteins. Anterograde cargo is captured into a COPII pre-budding complex with the activated GTPase sar1 and the inner coat sec23/24 heterodimer. Sar1 interacts directly with sec23, while the cargo is bound in several distinct pockets on the membrane-proximal surface of sec24 [1–4]. Recruitment of the outer coat layer, comprised of sec13/31 heterotetramers, positions a flexible proline rich region (PRR) loop of sec31 across the membrane-distal surface of sec23 and inserts residues into the sar1 active site, potentiating the sec23 GAP activity. Cyclical sar1 GTPase activity is required for cargo concentration [5]. Sec13/31 recruitment involves polymerization of at least 24 heterotetramers [4], triggering vesicle scission. COPII vesicles fuse homotypically to produce vesicular tubular clusters (VTCs), the primary site of cargo concentration [6–10]. After transport to the pericentriolar region, VTCs concentrate and fuse with Golgi cisternae [2].

While Ca^2+^ is well established as a required cofactor in evoked exocytosis, regulatory roles for Ca^2+^ in intracellular membrane fusions are still becoming clear. Various intracellular transport steps display requirements for or involvement of Ca^2+^ [11,12], including ER-to-Golgi transport [13], intra-Golgi transport [14–19], and endosome and lysosome trafficking and fusion [20–24]. On the other hand, Ca^2+^ does not seem to play a universal or mechanistically conserved role, and the secretory pathway may be a mosaic of Ca^2+^-dependent and -independent transport steps [25]. Recent work on ER-to-Golgi transport demonstrates that this step requires luminal Ca^2+^ stores at a stage following cargo biogenesis and folding/assembly, perhaps through release of Ca^2+^ into the cytoplasm where it binds and activates the vesicle budding, docking and/or fusion machinery [26,27]. Specific depletion of luminal calcium leads to significantly reduced transport and a buildup of budding and newly budded COPII vesicles and vesicle proteins [26,27].

Effector mechanisms by which Ca^2+^ modulates transport are not well understood. Calmodulin has been implicated in several transport steps [11,20,21,23,24], and Ca^2+^-dependent phospholipase A2 may regulate Golgi membrane dynamics [28]. The penta-EF-hand-containing (PEF) protein family are cytoplasmic calcium-dependent adaptors that have been implicated in many Ca^2+^-dependent cellular phenomena and may regulate ER-to-Golgi trafficking upon Ca^2+^ binding [29]. The PEF protein apoptosis-linked gene-2 (ALG-2) acts as a Ca^2+^ sensor at ER exit sites and stabilizes association of sec31 with the membrane when Ca^2+^ is present [30–33]. While it is clear that ALG-2 can affect ER export, the physiological conditions under which ALG-2 is rate-limiting have not been clarified. Furthermore, it is not clear whether ALG-2 binding to sec31 inhibits vs. promotes cargo export. In vitro studies found that purified ALG-2 attenuated budding in a Ca^2+^-dependent manner, and that ALG-2 binding to sec31A directly promoted sec31A-sec23 interactions [34]. Another in vitro study showed that purified ALG-2 inhibited COPII vesicle fusion, most likely by inhibition of vesicle uncoating preceding fusion [26]. Using intact cell approaches, one study found that ALG-2 depletion resulted in increased VSV-G transport [35], implying an inhibitory role for ALG-2, while another demonstrated that disrupting ALG-3/sec31A interactions inhibited VSV-G transport, implying a stimulatory role [27]. Furthermore, work on a presumed ALG-2 ortholog in yeast, Pef1p, demonstrated an inverse relationship, wherein Pef1p binding to the sec31 PRR was inhibited by Ca^2+^ and delayed coat recruitment to the membrane [36].

Although some of the apparent discrepancies described above may be explained by different experimental conditions, the issue of whether ALG-2 binding to sec31 has a stimulatory vs. inhibitory role remains a challenge. Here we took a different approach to investigating PEF protein function in ER-to-Golgi transport by investigating the role of peflin, a PEF family member that binds to ALG-2 and is presumed to regulate its activity. ALG-2 and peflin form cytosolic heterodimers that dissociate in response to calcium [37]; however, whether and how peflin regulates ALG-2/sec31A interactions and/or ER-to-Golgi transport has not been reported. In fact, no biological functions for peflin have been reported. We found that depletion of peflin significantly stimulated ALG-2 targeting to ERES and ALG-2/sec31A interactions and concomitantly stimulated ER-to-Golgi transport. Hence, peflin is a negative regulator of ALG-2/sec31A interactions and ER-to-Golgi transport under basal conditions.

## Results and Discussion

### Peflin depletion increases ER-to-Golgi transport

**Fig 1:**
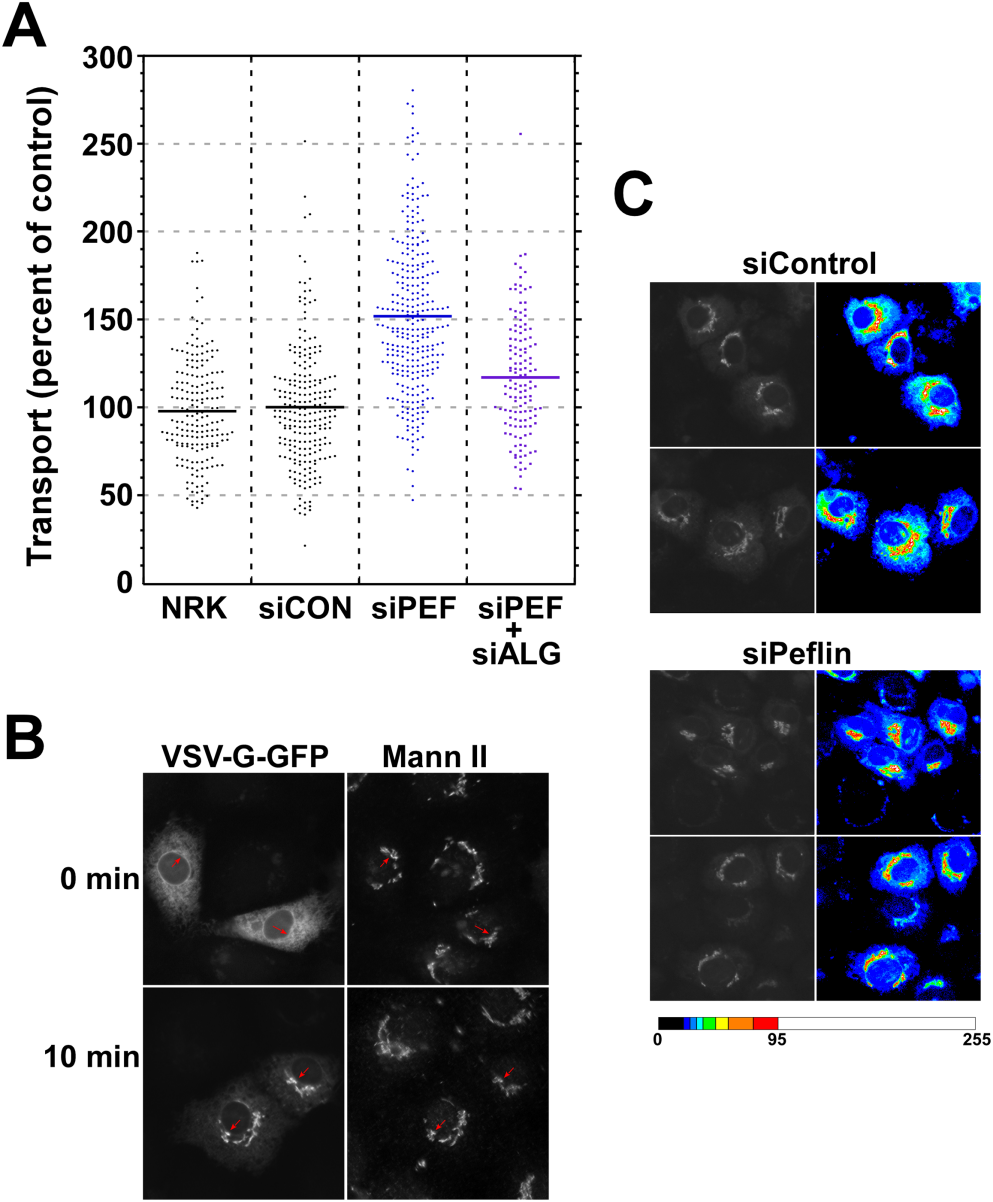
Peflin depletion accelerates ER-to-Golgi transport of VSV-G-GFP. NRK cells were either not transfected (NRK) or transfected with the indicated control (siControl), peflin (siPEF), or ALG-2 (siALG) siRNAs. Transport of VSV-G-GFP was conducted by shifting coverslips from 40 to 32°C medium for 10 minutes followed by immediate fixation and processing for immunofluorescence of GPP130 and mannosidase II (Mann II) to identify VSV-G-GFP in the Golgi. (A) Transport of VSV-G-GFP to the Golgi was quantified from random images using a transport index algorithm representing the ratio of Golgi to peripheral green fluorescence. The transport index of each individual cell is plotted. T-tests were performed: siControl vs siPeflin n = 519, p<0.0001; siControl vs siPeflin+ALG-2 n = 358, p<0.0001; siPeflin vs siPeflin+ALG-2 n = 425, p<0.0001. (B) Example widefield images of siControl cells showing increased concentration of VSV-G-GFP in the Golgi, and decreased reticular fluorescence, upon incubation at 32°C for 10 min. Arrows point to Golgi structures in VSV-G-GFP-transfected cells. Similar sets of images including the knockdown conditions are shown in S2 Fig. (C) Example widefield images typifying differences in VSV-G-GFP transport between siControl and siPeflin cells. siControl cells (top 4 blocks) exhibit a lower ratio of Golgi to peripheral GFP fluorescence compared to siPeflin cells (bottom 4 blocks). This lower ratio is very apparent in random quantitation of cells (A), but can also be detected by eye in grayscale images (left 4 blocks) and is apparent in heat-mapped grayscale images (right 4 blocks), where the Golgi fluorescence (white, orange, red and yellow) is surrounded by skirts of moderately intense reticular/vesicular fluorescence (green, blue-green and light blue) that becomes less intense (dark blue) near the edge of cells. Reticular/vesicular fluorescence adjacent to Golgi regions is elevated in siControl cells relative to siPeflin cells, consistent with a slower emptying of the ER. Legend to the heatmap is below, with pixel intensity values from 0 to 255 indicated. Golgi maximum intensities in the four heatmapped images ranged from 101 to 127.

To clarify the role of PEF proteins in secretion, we depleted peflin from normal rat kidney (NRK) cells using siRNA and measured ER-to-Golgi transport of the glycoprotein VSV-G-GFP ts045, a temperature-sensitive transmembrane cargo. Our morphological transport assay [27,38] involves fixation of live cells 10 minutes following shift of VSV-G-GFP to the permissive temperature and measurement of a morphological transport index comprised of the ratio of Golgi- to ER-localized VSV-G-GFP. As documented in Fig 1A–1C, peflin-depleted cell populations displayed strikingly increased ER to Golgi transport compared to untreated or control siRNA-depleted NRK cells. The mean transport index increased by 1.5-fold, corresponding to at least 50% faster ER-to-Golgi transport. This result indicates that under basal cellular conditions, peflin acts as an inhibitor, or negative regulator, of transport. The increased transport in peflin-depleted cells was observed for three indpendent and non-overlapping siRNAs for rat peflin (S1 Fig).

Knockdown of ALG-2 using siRNA was reported to have no effect on transport [32], or contradictory effects [27,35] that were small compared to experimental errors. Given the uncertainty of ALG-2 function together with the propensity of ALG-2 and peflin to form heterodimers [37], it was difficult to predict how co-depletion of ALG-2 would affect the increased transport observed in the absence of peflin. As shown in Fig 1A, double-depletion of peflin and ALG-2 together resulted in ablation of most of the increased transport attributed to peflin depletion. Depletion of ALG-2 in the absence of peflin significantly decreased transport, implying that, at least in the absence of peflin, ALG-2 plays a *positive* regulatory role. It also implies that the mechanism by which peflin normally suppresses transport requires the presence of ALG-2. Single knockdown of ALG-2 was similar to control but very difficult to interpret (see below).

**Fig 2:**
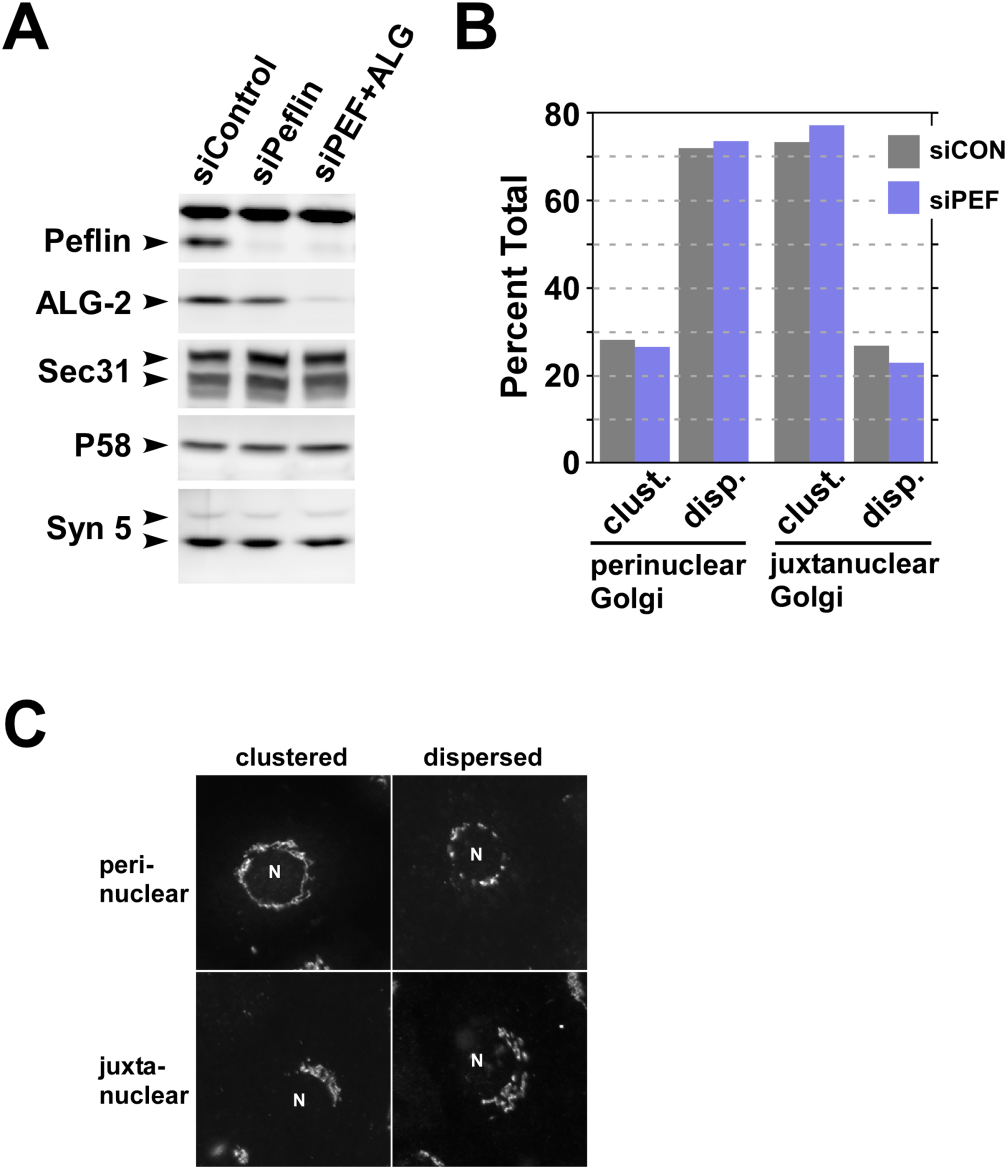
Demonstration of knockdown efficiencies and lack of Golgi disruption. (A) Cell lysates from the experiments documented in Fig 1 were immunoblotted to detect the proteins indicated along the left edge of the image blocks. Sec31A has multiple splice isoforms resulting in multiple compound bands; syntaxin 5 is found in a long and short form due to alternative translation initiation sites. (B) Mannosidase II and GPP130 labeling of siControl and siPeflin cells were scored by visual pattern recognition into two general Golgi morphologies, perinuclear or juxtanuclear, indicating whether the Golgi abuts against more or less than one continuous half of the perimeter of the nucleus. Each Golgi was then further classified as either clustered or dispersed. Approximately 150 cells from each experimental group were classified, and clustered vs. dispersed Golgis were expressed as a percentage of the total within their general morphology type, perinuclear or juxtanuclear. There were no noted differences in Golgi dispersion between the populations. The ratio of perinuclear to juxtanuclear Golgis was approximately 0.8:1 for both populations. (C) Example images of the 4 types of Golgi morphology scored in (A). An “N” marks the nucleus in the example cells.

To interpret the functional results it was important to establish the effectiveness and specificity of the siRNA treatments. As shown in Fig 2A, peflin siRNA was very effective (~95%) at eliminating peflin. In some experiments, a slight decrease in ALG-2 was also observed, perhaps due to increased turnover of ALG-2 when not present in the peflin/ALG-2 heterodimer [39]. This coincident decrease in ALG-2 was variable and never exceeded 40%. In the double knockdown condition, peflin depletion remained ~95% complete and ALG-2 was depleted by ~90%. Immunoblotting for several other COPII and ERES-related proteins established specificity of the depletions. We noted that single depletion of ALG-2 greatly destabilized peflin and resulted in almost the double knockdown condition (data not shown). Under this condition, the ~10% residual ALG-2 may have a greatly increased ratio of ALG-2 homodimer to ALG-2:peflin heterodimer. For these reasons, we do not show nor attempt to interpret an ALG-2 single knockdown.

As a further evaluation and control for the peflin knockdown phenotype, we quantitated the distribution of Golgi elements in the cells. Changes in organization of the secretory pathway could alter transport rates without directly affecting the trafficking machinery. Golgi marker distribution is a sensitive indicator of early secretory organization because Golgi formation and homeostasis is closely tied to ERES distribution, recycling of secretory machinery, and microtubule-based transport. However, as shown in Fig 2B and 2C, the overall distribution of Golgi markers did not change in peflin-depleted cells with respect to the proportion of cells with a compact vs. dispersed Golgi labeling pattern. This adds additional confidence that the increase in transport caused by peflin depletion was caused by direct change(s) in transport machinery rather than a spatial re-organization of organelles.

### Peflin depletion increases ALG-2 targeting to ERES and interactions with sec31A

**Fig 3:**
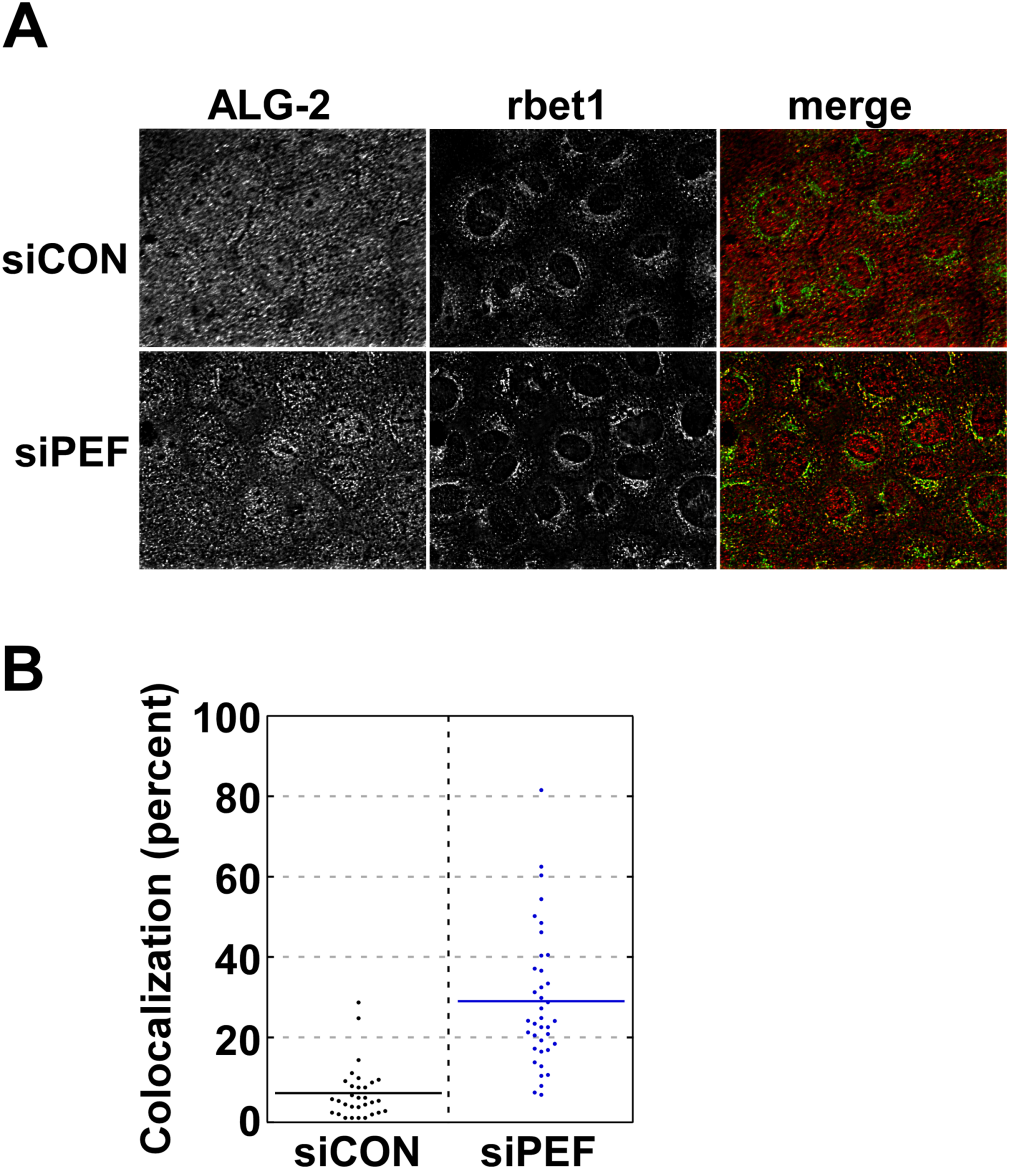
Peflin depletion causes increased targeting of ALG-2 to COPII membranes. (A) NRK cells transfected with the indicated siRNA were fluorescently labeled with anti-ALG-2 antibody and cy3, or anti-rbet1 antibody (ERES and early VTC marker) and cy5. Maximum intensity projections from deconvolved widefield z-stacks are displayed in grayscale along with the corresponding pseudocolor merged panel. Increased focal and decreased diffuse labeling of ALG-2 and an increased quantity of yellow in the merged images can be observed in siPeflin cells. (B) Quantitation of multiple images. Colocalization was defined as the proportion of the total ALG-2 labeling that was coincident with rbet1 labeling, calculated for each cell individually in randomly selected fields. A T-test was performed between the siControl group and siPeflin: n = 69, p<0.0001.

Our next goal was to identify changes in ERES or COPII vesicle molecules that could help explain peflin's regulatory role in transport. Since peflin binds ALG-2 and ALG-2 partially localizes to ERES, we asked whether peflin depletion changes ALG-2 targeting to early secretory membranes. ALG-2 displays a varied localization, with pools present in the nucleus, the cytosol, and ERES [30,32]. As shown in Fig 3A, top row, in mock-depleted NRK cells, endogenous ALG-2 displays the expected mixed localization pattern. Some ALG-2 is localized to cytoplasmic punta that co-localize with rbet1, a transmembrane ER/Golgi SNARE that localizes to ERES, COPII vesicles, and VTCs [40]. As seen in the bottom row on the other hand, in peflin-depleted cells ALG-2 is less diffuse and significantly more ALG-2 is localized in cytoplasmic puncta that co-localize with rbet1. Co-localization of ALG-2 with rbet1 was quantified in ~70 cells in Fig 3B; while there was <10% co-localization in mock-depleted cells, there was ~30% co-localization in peflin-depleted cells. Since there was no significant change in rbet1 distribution caused by peflin knockdown, we conclude that the increased co-localization was caused by increased targeting of ALG-2 to rbet1-positive membranes.

**Fig 4:**
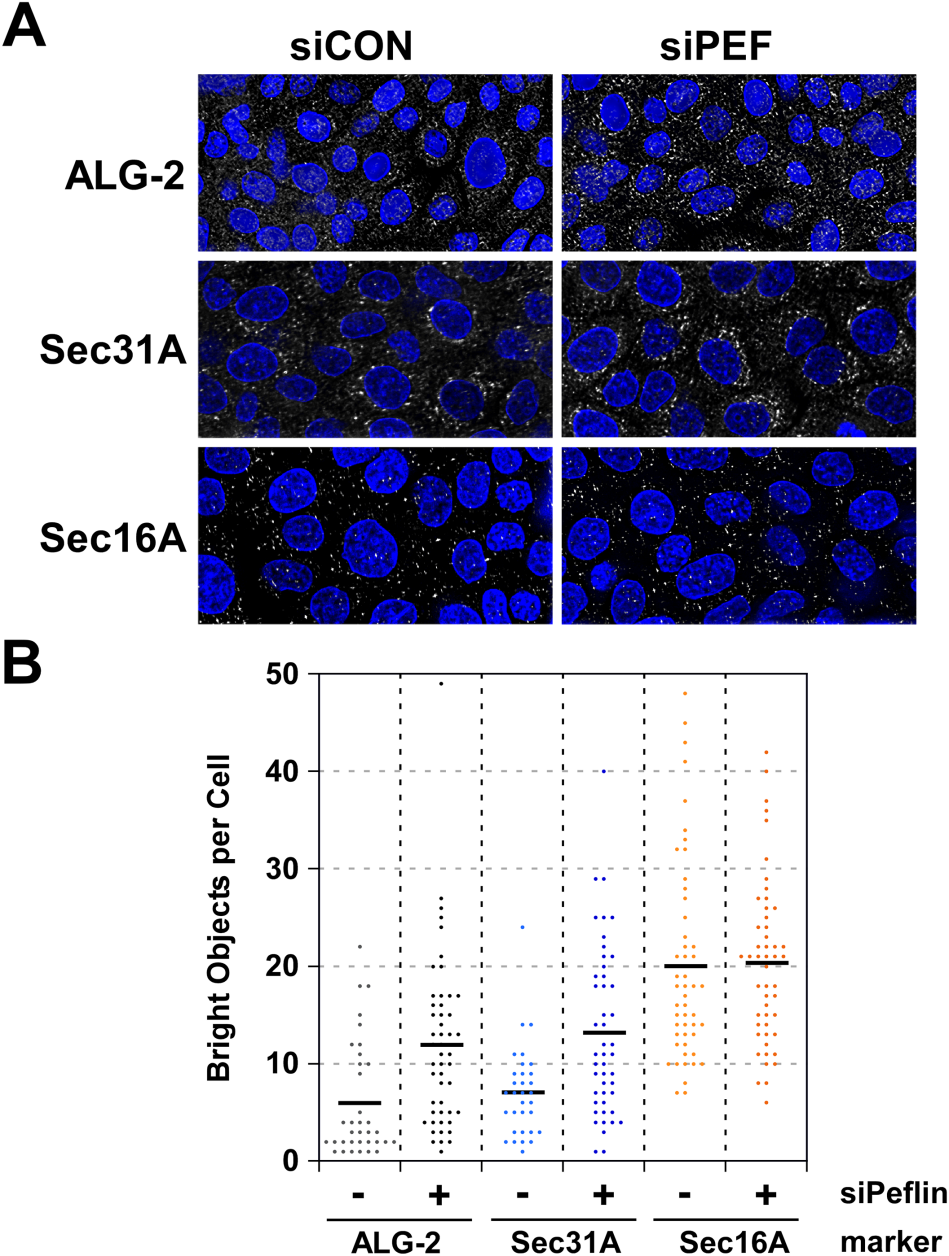
Peflin depletion increases punctate subcellular localization of ALG-2 and sec31A, but not sec16A. (A) NRK cells transfected with the indicated siRNA were labeled with anti-ALG-2, anti-sec31A, or anti-sec16A antibodies, a cy3-conjugated secondary antibody, and dapi. Maximum intensity projections from deconvolved widefield z-stacks of the ERES proteins are displayed in grayscale merged with the corresponding dapi panel. (B) Quantitation of 4–5 fields of ~10 random cells per field, for each condition. A consistent intensity threshold was used to automatically capture and count bright cytoplasmic objects. A T-test between the siControl and siPeflin group was performed for each labeled protein: ALG-2, N = 85, p = 0.0003; Sec31A, N = 79, p = 0.0001; Sec16A, N = 100, p = 0.866.

We also tested whether depletion of peflin strengthened ERES through increased targeting of other peripheral membrane protein components. As shown in Fig 4A and quantitated in Fig 4B, peflin siRNA caused an increase in bright cytoplasmic spots in cells immuno-labeled to detect ALG-2 and sec31A, but no detectable change in sec16A labeling. Increased spotty labeling likely represents increased targeting from less visible cytosolic pools since peflin depletion did not alter either ALG-2 or sec31A expression (Fig 2A) enough to explain the increased punctate labeling. Importantly, the increased targeting of ALG-2 and sec31A to these puncta correlates with increased ER-to-Golgi transport in peflin knockdown cells.

**Fig 5:**
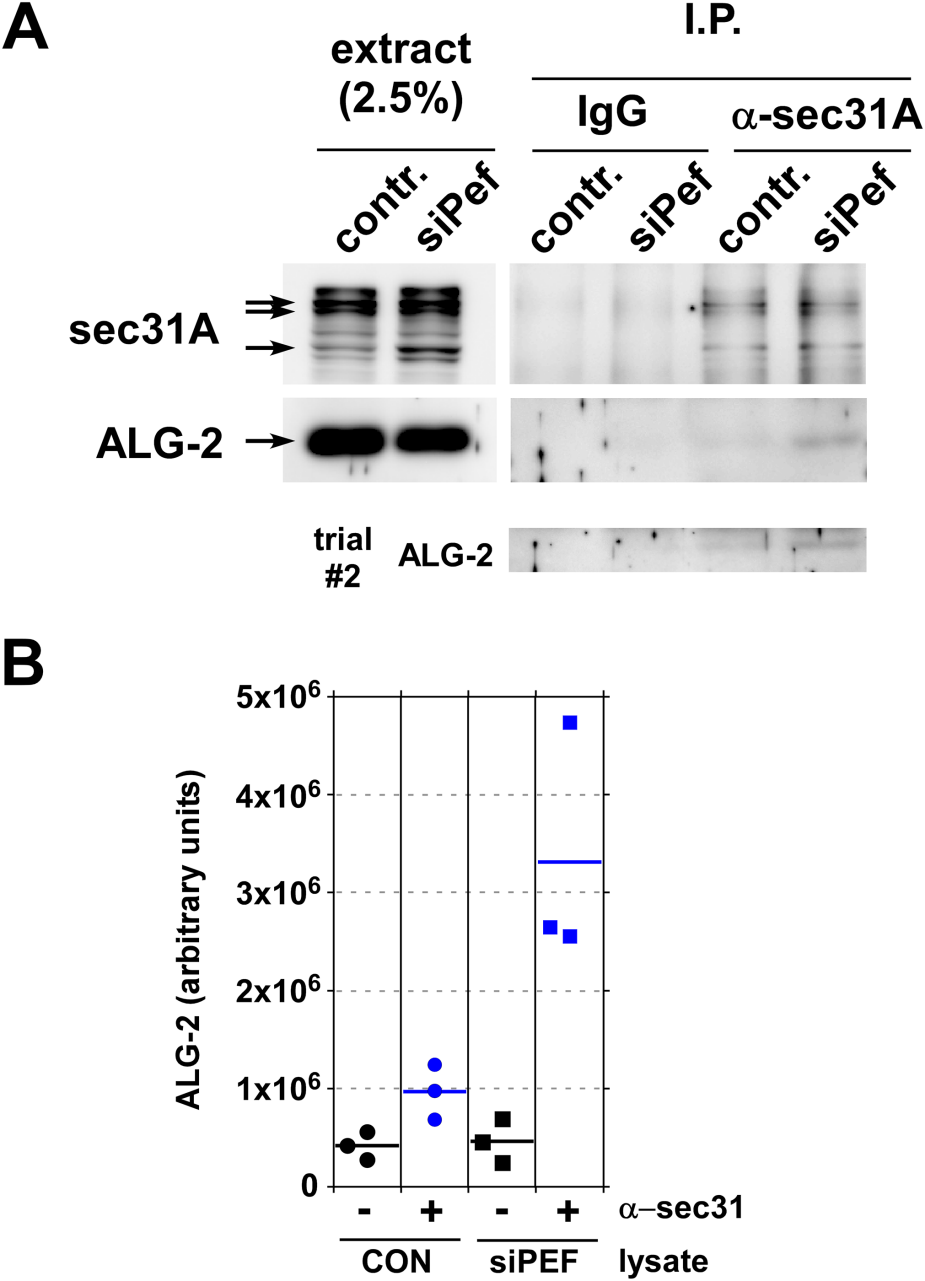
Peflin depletion causes increased ALG-2 interactions with sec31A. (A) Whole cell CHAPS extracts of control and peflin knockdown NRK cells were subjected to immunoprecipitation with IgG- or anti-sec31A-conjugated protein A-Sepharose beads. Beads were eluted with SDS and samples were analyzed by SDS-PAGE and immunoblotting to detect sec31A and ALG-2. Left lanes show input extracts at the indicated load level relative to the immunoprecipitate. Immunoprecipitation of endogenous sec31A was inefficient, resulting in weak ALG-2 co-immunoprecipitation signals; for this reason, the ALG-2 immunoblot from an independent repetition of the experiment (trial #2) is shown below. (B) Quantitation of three completely independent repetitions of the co-immunoprecipitation experiment. ALG-2 specifically co-precipitated with sec31A under control conditions, and this co-precipitation increased in peflin-depleted cells.

ALG-2 is known to interact with sec31A at ERES, where it stabilizes the outer shell on the membrane [32] and increases co-assembly of inner and outer shell [34]. To test whether peflin depletion activates this mechanism, we immunoprecipitated endogenous sec31A complexes and tested whether endogenous ALG-2 co-immunoprecipitated. As shown in Fig 5A, top, sec31A inefficiently but specifically immunoprecipitated with our affinity-purified polyclonal antibody. Note that the sec31A signal is heterogeneous due in part to multiple splice isoforms. In NRK cell extracts all of the bands in the region marked by arrows disappear when cells are treated with a single sec31A siRNA [27]. Specificity of the immunoprecipitation with anti-sec31A beads (“α-sec31A” lanes) is demonstrated by the lack of bands in lanes treated with non-immune IgG beads ("IgG" lanes) and with anti-sec31A beads plus a sec31A 20-amino synthetic peptide comprising the antibody antigen (data not shown). As shown in Fig 5A, middle panels, endogenous ALG-2 weakly co-precipitates with sec31A, in agreement with the relatively small co-localization to ERES observed by microscopy (Fig 3B). In peflin knockdown cells, however, the ALG-2 co-immunoprecipitation signal is significantly stronger. As can be seen in the quantification of several experiments in Fig 5B, about 3-fold more ALG-2 co-precipitates with sec31A in peflin-depleted cells. Since the ALG-2-sec31A interaction is believed to occur at ERES [32], this experiment demonstrates that in the absence of peflin, more ALG-2 is targeted to ERES where it binds sec31A.

**Fig 6:**
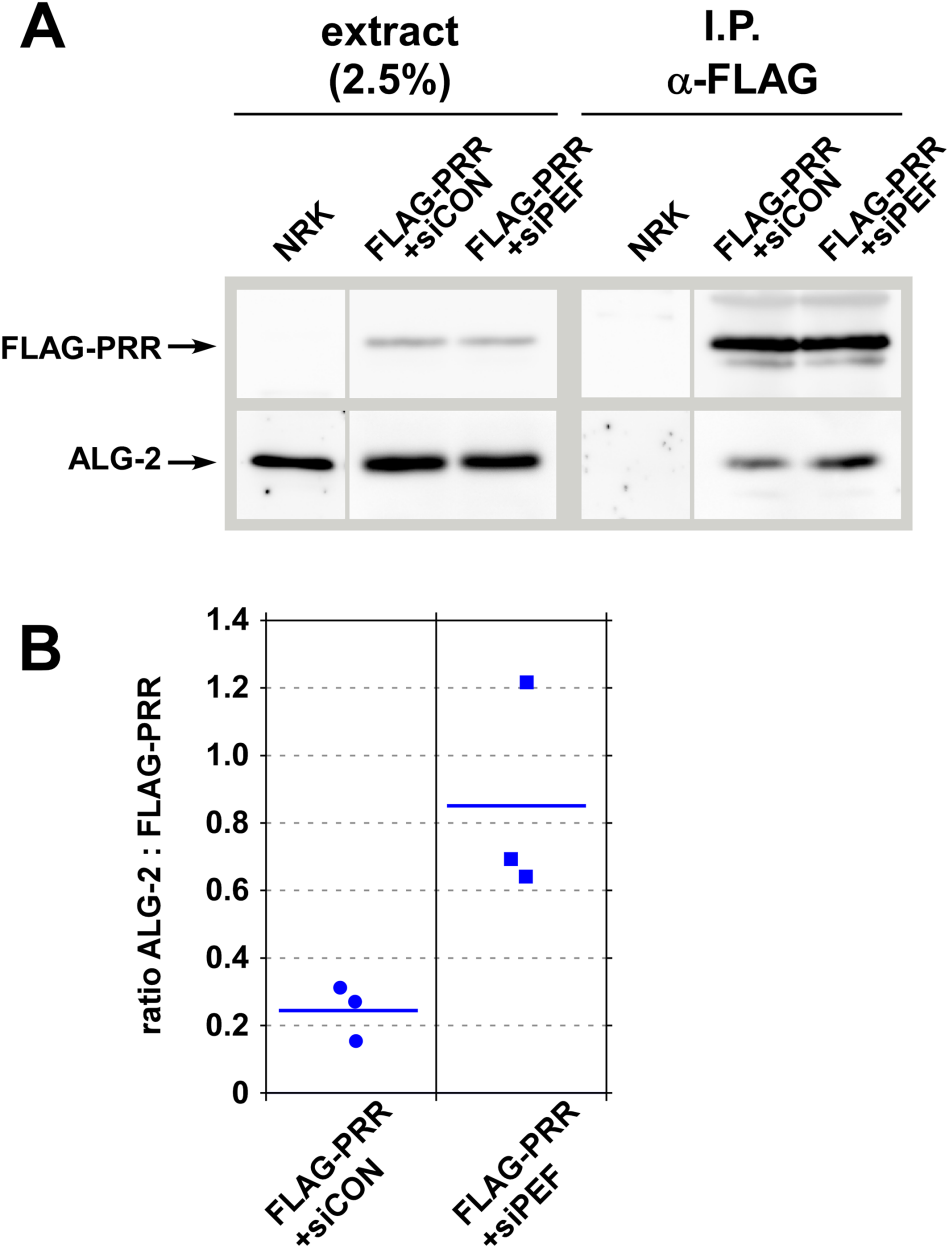
Peflin depletion causes increased ALG-2 interactions with the sec31A PRR region. (A) NRK cells were either not transfected or transfected with a FLAG-PRR construct (human amino acids 800–1113) and either control or peflin siRNA. Whole cell CHAPS extracts were then subjected to immunoprecipitation with anti-FLAG-conjugated protein A-Sepharose beads. Beads were eluted with SDS and samples were analyzed by SDS-PAGE and immunoblotting to detect FLAG and endogenous ALG-2. Left lanes show input extracts at the indicated load level relative to the immunoprecipitate. (B) Quantitation of three independent repetitions of the co-immunoprecipitation experiment. ALG-2 specifically co-precipitated with FLAG-PRR under control conditions, and the co-precipitation increased in peflin-depleted cells. Yield of ALG-2 in the precipitates in arbitrary units was expressed as a ratio to the yield of FLAG-PRR in arbitrary units. Quantifications were performed on exposures of the blots that gave similar, unsaturated band intensities in siPEF I.P. lanes.

To further specify the effects of peflin knockdown on ALG-2-sec31A interactions, we tested whether peflin depletion increases the propensity of ALG-2 to bind to the sec31 PRR region in NRK cells. A FLAG-PRR 800–1113 construct containing just the sec31A PRR region was co-transfected with either the control or peflin siRNAs. As shown in Fig 6A and quantitated in Fig 6B, peflin depletion increased co-precipitation of endogenous ALG-2 with the PRR construct, indicating that ALG-2 PRR-binding activity increased in peflin-depleted cells.

**Fig 7:**
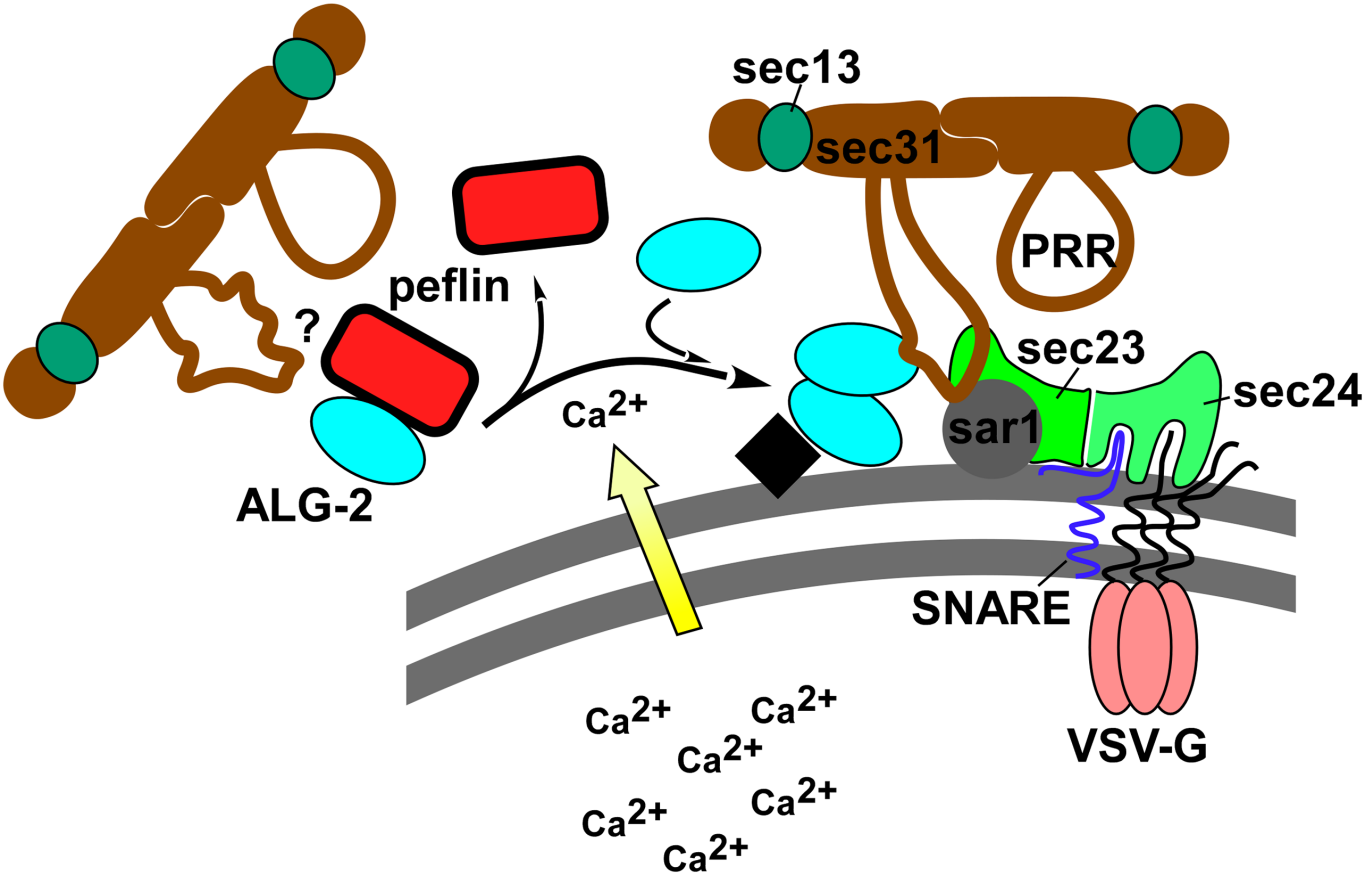
Model for peflin regulation of ER-to-Golgi transport. Peflin exists as a heterodimer with its binding partner ALG-2. In the presence of calcium peflin dissociates, allowing ALG-2 to homodimerize and undergo Ca^2+^-dependent binding to the proline rich region (PRR) of sec31A. ALG-2 binding to the PRR activates the PRR for interactions with inner shell components sar1 and sec23, which stabilizes the coat assembly on the membrane. These events may increase ER-to-Golgi transport. However, we cannot eliminate the possibility that peflin may also or instead regulate transport through unknown interactions. It is also not known whether the peflin-ALG-2 heterodimer binds the PRR region and affects its activity ("?" to left of heterodimer). The black square represents a hypothetical docking site for ALG-2 at the ERES.

In summary, in the absence of peflin, more ALG-2 was targeted to ERES (Fig 3), more sec31A was targeted to ERES (Fig 4), and ALG-2-sec31A interactions increased (Fig 5) through ALG-2 binding to the PRR region (Fig 6). It is important to note that although peflin knockdown causes both increased ALG-2-dependent transport and ALG-2 targeting to ERES where it binds the sec31A PRR, it cannot be formally concluded that ALG-2 binding sec31A, per se, *causes* the increased transport. However, until an entirely different mode of peflin action is discovered, it is safe to conclude that the mechanism of action of peflin in transport is closely related to ALG-2. Furthermore, the demonstrated peflin modulation of ALG-2/sec31A interactions at ERES would represent a strikingly consistent mechanism for the transport effect. Fig 7 presents our working model of peflin regulation of secretion, wherein release of ALG-2 from its binding partner peflin causes it to translocate to ERES and bind sec31A. Calcium has been shown to cause both dissociation of the peflin/ALG-2 heterodimer [37] and stimulate ALG-2 homodimer binding to sec31A [31], so one attractive possibility is that the peflin depletion studies presented here mimic what happens upon luminal calcium release in the cell. However, more information about peflin's other potential binding partners and any potential calcium-dependent regulation of PEF protein expression levels would be required to refine this model.

Another remaining question is why in vitro reconstitution studies of budding and fusion so far have only reported inhibitory roles for ALG-2, while in the present study increased ALG-2/sec31A interactions accompany increased, rather than decreased, ER export. One possibility is that purified recombinant ALG-2 is not fully functional. Another potential explanation could be that in vitro systems do not recapitulate the timed cellular sequence of necessary positive and negative effects for optimal secretion. Budding must be inhibited until cargo is loaded and then promoted; vesicle uncoating must be inhibited until after budding, and then promoted, etc. This can create seemingly counterintuitive roles where inhibitors play required or permissive roles. To take one relevant example, a required Ca^2+^ effector for endosome fusion is a Ca^2+^-dependent rab11 GAP, i.e. an inhibitor of a rab that is required for fusion [24]

## Materials and Methods

### Antibodies –

Rabbit polyclonal anti-sec31 was produced previously in rabbits against a synthetic peptide [26]. Monoclonal anti-rbet1 and polyclonal anti-syntaxin 5 antibodies were described before [40]. Rabbit polyclonal anti-p58 was a kind gift from Dr. Jaakko Saraste (University of Bergen, Norway). Rabbit polyclonal anti-ALG-2 was a kind gift from Dr. Masayuki Komada (Tokyo Institute of Technology). Rabbit anti-GPP130 antibody was purchased from Covance (product PRB144C). Mouse monoclonal anti-mannosidase II antibody was purchased from Covance Research Products (product MMS-110R-200). A rabbit anti-peflin polyclonal antibody was purchased from Novus (product NBP1-31613). Rabbit anti-sec16A (product NB100-1799) was from Novus Biologicals. Secondary antibodies were FITC-, Cy3-, AMCA-, or cy5-conjugated and purchased from Jackson ImmunoResearch Laboratories (West Grove, PA).

### siRNA knockdown of peflin and ALG-2

NRK cells were electroporated with 0.6 μM siRNA (Ambion, Life Technologies Corp.). For double knockdowns, cells were electroporated with 0.43 μM of each siRNA. After 2–3 days of normal growth at 37°C, the cells were resuspended and re-electroporated, this time with a combination of the siRNA plus 7.5 μg of VSV-G-GFP plasmid, and the cells were allowed to recover and grow on coverslips at 40°C. Twenty-four hours later, the cells were either lysed directly in CHAPS extraction buffer for quantitative immunoblotting or immunoprecipitation analysis, or else processed for transport assays as described below. Peflin siRNA s150975 had the following sense strand sequence: 5' -GCCUCAUGAUGAUAAACAU-3' (lacking Silencer Select chemical modifications) and consistently achieved ~95% knockdown. ALG-2 siRNA had the following sense strand sequence: 5' -GGAGCGGAGUGAUUUCAGA-3' (containing Silencer Select chemical modifications) and consistently achieved ~90% knockdown. Control siRNAs were custom synthesized non-targeting siRNAs from the same manufacturer. Immunoblotting of cell lysates (prepared as described below in the immunoprecipitation section) enabled validation of knockdown efficiencies for each siRNA experiment that was functionally analyzed.

### ER-to-Golgi transport assay

NRK cells were maintained in DMEM high glucose containing 10% fetal calf serum and penicillin-streptomycin. Suspensions of NRK cells were electroporated with siRNA and VSV-G-GFP as described above, and plated on poly-lysine coated coverslips. After 24 hours of VSV-G-GFP expression at 40°C, the cells were either fixed by dropping coverslips directly into fixative, or dropped into to 6-well chambers containing pre-equilibrated 32°C medium for 10 min, then transferred to fixative. Coverslips were fixed with 4% paraformaldehyde containing 0.1M sodium phosphate (pH 7) for 30 min at room temperature and quenched twice for 10 min with PBS containing 0.1M glycine. Fixed cells were treated for 15 min at room temperature with permeabilization solution containing 0.4% saponin, 1% BSA, and 2% normal goat serum dissolved in PBS. The cells were then incubated with primary antibodies diluted in permeabilization solution for 1 hr at room temperature. Next, coverslips were washed 3x with permeabilization solution and incubated 30 min at room temperature with Cy3- or Cy5-conjugated anti-rabbit and -mouse and secondary antibodies. After the secondary antibody incubation, coverslips were again washed 3x using permeabilization solution and mounted on glass slides using Slow Fade Gold antifade reagent (Invitrogen) and the edges sealed with nail polish. Slides were analyzed using a 40x objective on a Nikon E800 microscope with excitation and emission filter wheels (Chroma tech), Hamamatsu Orca 2 camera and Nikon Z-drive, automated using OpenLab 5.5 software (Improvision). For transport index assays, typical images collected for each field of cells were VSV-G-GFP (FITC filters), anti-GPP130 (Cy3 filters) and anti-Mannosidase II (Cy5 filters).

Morphological quantitation of ER-to-Golgi transport was accomplished as described before [27,38] but using larger sample sizes. Briefly, images were collected in a consistent manner with regard to cell morphology, protein expression levels and exposure. After choosing a fixed exposure time for each color channel that would accommodate the vast majority of cells, we avoided any cell whose intensity values in any color exceeded the saturation value of our camera. A single widefield image plane was collected for each color channel for each field of cells randomly encountered; image deconvolution was not performed. Images were exported as 14-bit Tifs and analyzed using Fiji open source image software with automation by a custom script. On the GFP image plane, the user defines the minimal rectangular ROI encompassing the cell to be analyzed, making certain that some dark, extracellular regions are represented along at least one edge of the ROI. This ROI is then isolated in a separate window and two parameters extracted; background, which represents the highest pixel intensity among the lowest 0.100 percentile of nonzero pixel values; and Golgi maximum, which represents the mean intensity of the pixels in the 99.990 percentile and above but excluding the highest pixel. The user checks that these brightest pixels are in fact within the Golgi as defined on the Mannosidase II or GPP130 image planes. The user then sequentially defines 3 small square ROIs within vesicular/reticular regions adjacent to the nucleus but clearly distinct from the Golgi area and avoiding thin areas of cytoplasm near the edge of the cell. The ER mean was extracted as the mean of the three mean pixel intensities of these ROIs. Transport index was then calculated for each individual cell as (Golgi maximum-background) / (ER mean-background). The cell was then numbered on the image to avoid re-counting, and all extracted parameters written to an appendable output file along with the cell number so that the data was traceable. The user then defines another cell from the image or opens another image. Using this method, the user quantitates about 50 cells per hour.

Once transport indices have been obtained for all conditions in an experiment, each value is subtracted by the mean transport index value for cells that were fixed directly from 40°C without a transport incubation at 32°C (typically a value between 1.0 and 1.5) to generate the net transport index. Net transport indices are then normalized to the mean siControl value for the particular experiment, prior to plotting and comparison between experiments. Each result reported here was obtained in at least three separate experiments, though the data shown in Fig 1 are from a single replicate.

### Colocalization analysis

Coverslips were fixed and labeled for immunofluorescence as described above but using rabbit anti-ALG-2, mouse anti-rbet1, anti-rabbit-Cy3, and anti-mouse-Cy5 antibodies. Slides were analyzed as above but using a 60x objective. For each field of cells, 21 image planes were captured from each color channel in 200 nm increments. These image stacks were deconvolved using Huygens Essential Widefield software (Scientific Volume Imaging, Hilversum, The Netherlands). Example images in Fig 3A represent maximum intensity projections of five consecutive layers near the center of the stack. For quantification of co-localization, deconvolved maximum intensity projections from random fields of siControl and siPeflin cells were background subtracted by defining a dark extracellular area of the image as zero. Images were then thresholded at a value 25-times the intensity of dull, cytoplasmic areas to define ALG objects and rebt1 objects. Boolean image operations were performed to determine the number of pixels within ALG-2 objects that were coincident with rbet1-positive pixels. This number of pixels was then divided by the total number of ALG-positive pixels, calculated for each cell individually within each field of randomly selected cells. Quantitation of images in Fig 4 used similar methods except that number of bright objects rather than overlap of bright objects was quantitated and thresholding to detect objects used a factor of 5-times the intensity of well-defined but faint spots.

### Immunoprecipitation experiments

For the experiment in Fig 5, anti-sec31A beads and non-immune IgG antibody beads were prepared by immobilization of purified antibodies onto protein A-Sepharose Fastflow beads at a concentration of 2 A_280_ units per ml of packed beads followed by covalent conjugation with dimethyl pimelimidate. NRK cells were transfected twice with control or peflin siRNA as described above. 24 hrs after the second transfection, cells were washed with ice-cold PBS and treated with 0.5 ml per 15 cm plate of CHAPS lysis buffer (20 mM Tris-HCl, pH 7.4, 150 mM KCl, 10 mM CHAPS, 2 μg/mL leupeptin, 4 μg/mL aprotinin, 1 μg/mL pepstatin A, 0.2 mM AEBSF). Cells were scraped from the plate in the CHAPS lysis buffer, Dounce homogenized, and then rotated end-over-end in microcentrifuge tubes for 1 hr at 4°C before centrifugation at 15,000 x g and snap-freezing the supernatant in small aliquots. On the day of an immunoprecipitation, extracts were thawed on ice and centrifuged at 100,000 x g for 20 min to remove precipitates immediately before use. All plastic microcentrifuge tubes used in the immunoprecipitation were preblocked with 5 mg/ml BSA in CHAPS lysis buffer. 50 μl of extract was used to suspend 7.5 μl of packed beads, the suspension of which was rotated end-over-end in the cap (sealed with Parafilm) of a 0.5 ml microcentrifuge tube for 2 hours at 4°C. After removal of the Parafilm seal, the beads were pelleted and washed three times with 500 μl of ice-cold CHAPS lysis buffer. Final aspirated bead pellets were resuspended to 25 μl with room temperature non-reducing SDS sample buffer, pelleted a final time, and the bead-free supernatant was adjusted to 10% beta-mercaptoethanol and boiled. Immunoblotting of immunoprecipitates and the starting extracts followed established techniques. The experiment in Fig 7 used similar methods except that 100 μl of extract was used per immunoprecipitation reaction and the antibody beads employed were commercial anti-FLAG beads (Sigma-Aldrich catalog number A2220).

## Supporting Information

S1 Fig Effects on ER-to-Golgi transport and peflin expression of three independent and non-overlapping siRNAs. (A) Each siRNA was used to transfect NRK cells and transport was analyzed as in Fig 1. (B) Immunoblotting of cells used in the transport experiments indicated that all three siRNAs silenced peflin, although siRNA 216986 was less effective than the other two. siRNAs were purchased from Ambion and are referred to by their Ambion ID numbers. The siRNA s150975 is the one used in other figures of the manuscript and described in the Methods. T-tests indicated the significance of siCON vs. each of the peflin siRNAs: 216986, N = 303, p<0.0001; 216987, N = 259, p<0.0001; s150975, N = 236, p<0.0001.
(PDF)

S2 Fig Example images of control and knockdown cells at two transport timepoints. Images demonstrate the same features as shown for control cells in Fig 1B, but include the knockdown conditions as well. In this set of images, the Golgi is marked with GPP130 instead of Mannosidase II. Red arrows point to the Golgi area in VSV-G-GFP-expressing cells.
(PDF)
