# Supplementary figures and images for: Penta-EF-Hand Protein Peflin Is a Negative Regulator of ER-To-Golgi Transport
Source: PLoS One. 2016 Jun 8;11(6):e0157227. doi: 10.1371/journal.pone.0157227 (PMC4898701; doi:10.1371/journal.pone.0157227)

**A**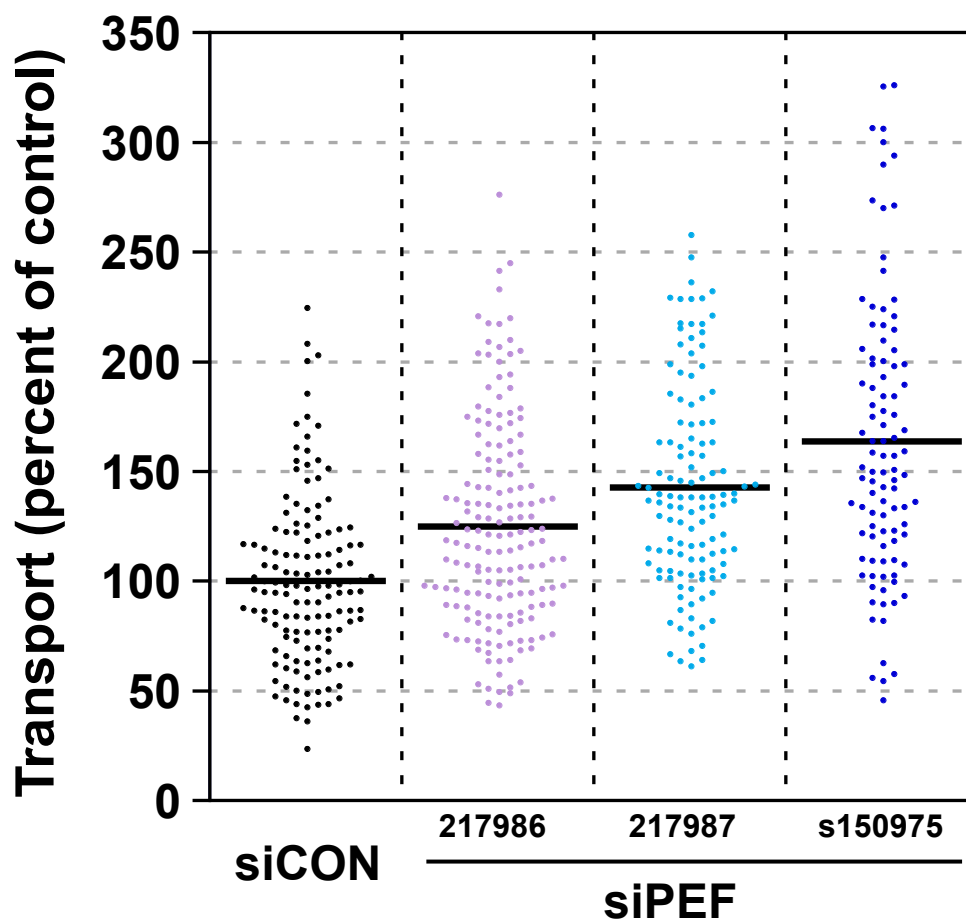**B**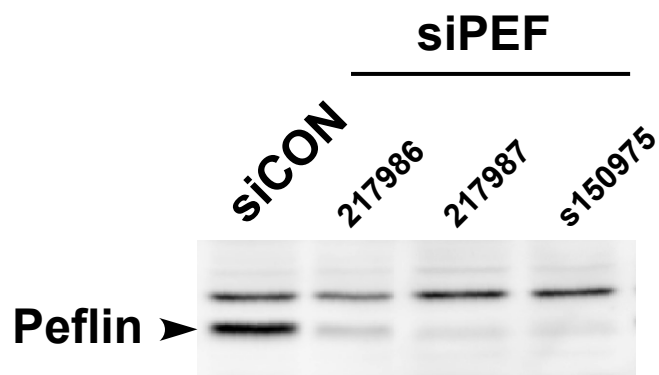

Supplement: S1 Fig — (A) Each siRNA was used to transfect NRK cells and transport was analyzed as in Fig 1. (B) Immunoblotting of cells used in the transport experiments indicated that all three siRNAs silenced peflin, although siRNA 216986 was less effective than the other two. siRNAs were purchased from Ambion and are referred to by their Ambion ID numbers. The siRNA s150975 is the one used in other figures of the manuscript and described in the Methods. T-tests indicated the significance of siCON vs. each of the peflin siRNAs: 216986, N = 303, p<0.0001; 216987, N = 259, p<0.0001; s150975, N = 236, p<0.0001. (PDF) [file pone.0157227.s001.pdf]

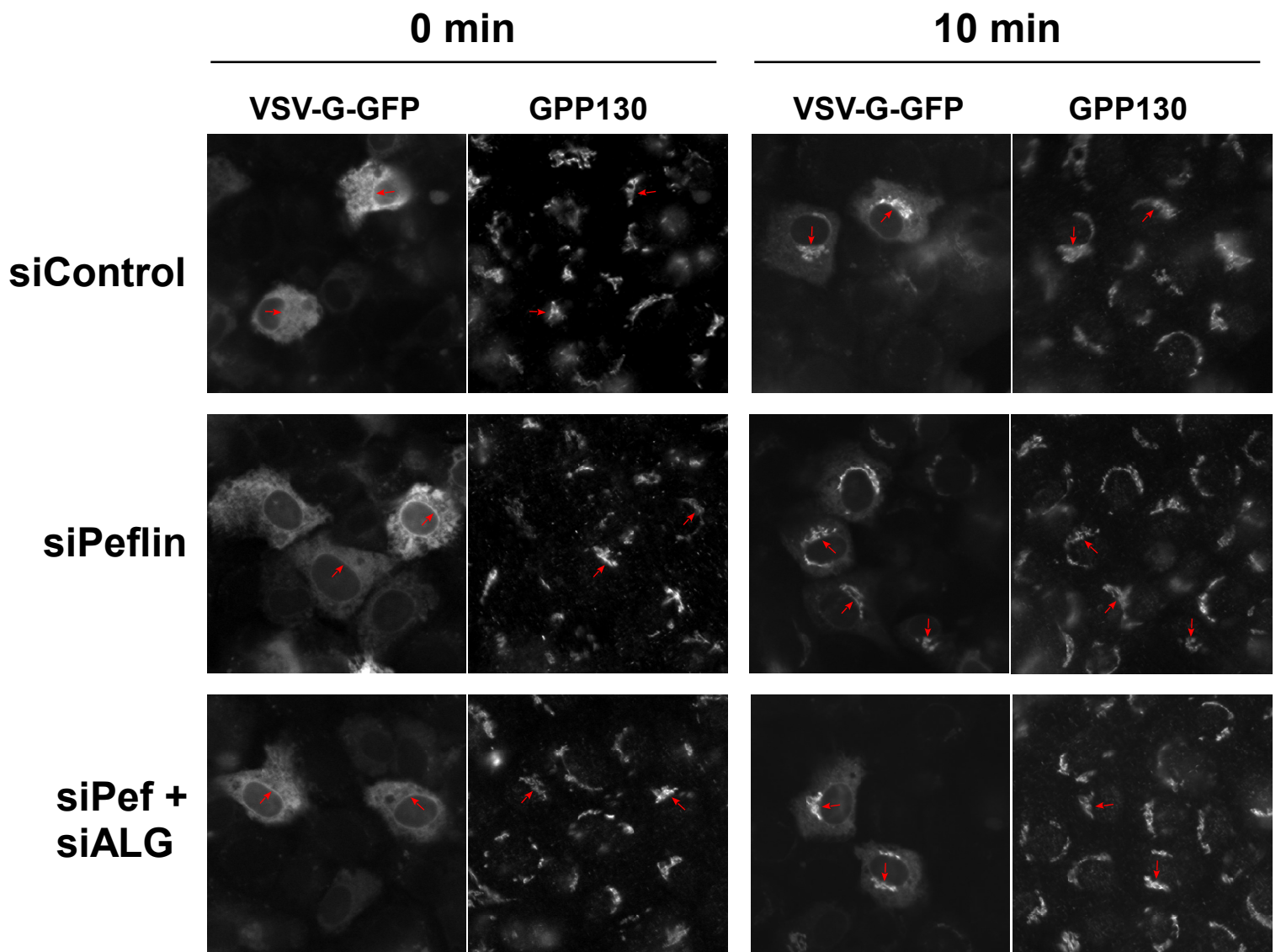

**S2 Fig.**

Supplement: S2 Fig — Images demonstrate the same features as shown for control cells in Fig 1B, but include the knockdown conditions as well. In this set of images, the Golgi is marked with GPP130 instead of Mannosidase II. Red arrows point to the Golgi area in VSV-G-GFP-expressing cells. (PDF) [file pone.0157227.s002.pdf]
